# The Dentist’s Duty to His Profession

**Published:** 1864-09

**Authors:** C. A. Marvin


### THE DENTIST'S DUTY TO HIS PROFESSION.

BY DR. C. A. MARVIN.

Much has been written bearing directly or indirectly upon this general subject. Experienced practitioners have uttered in the ears, and have written for the perusal, of students, their ideas of the Dentist's duty. These ideas, largely drawn from the personal experience of their authors, are most valuable, and go to form an important part of the great volume in process of preparation, containing and setting forth practical rules for practical men.

By the aid of these, and the improving effect of daily practice, what may we not hope for, when another decade shall have passed away, and the clearer conceptions of what is and what is not *duty* shall find utterance in well-chosen words from the lips of earnest faithful men, or shall stand imperishable, upon the printed page? Whoever of us shall live to see that period, may look for such accessions to our Dental Literature, as to day would appear incredible:—may expect to see our profession elevated to a position not inferior to that of, what are now termed, "the learned professions."

It has occurred to me, Mr. President and gentlemen, that it would not be inappropriate at this time, to give a little thought to that particular branch of this general subject of the Dentist's duty, named in the title of my paper, viz.: *the duty of Dentists to their profesion.*

You will perceive, that I have limited my line of thought to one direction, leaving all others, or any other, to be treated of at some future time.

What is the Dentist's duty *to his profession?* and how does this duty affect other duties in his life? are the two questions I propose to consider in this paper.

1st. What is the Dentist's duty to his profession?

Not his duty to his patient! not to the world! not to himself! his duty to his *profession*: *that* is my inquiry, and let me keep right there till I state my view of it.

I answer then in this one brief sentence: The Dentist's duty to his profession is *to seek to elevate that profession, by every means in his power.*

To do this, he must become acquainted with it; nay he must *know it through and through.* This necessitates study. The Dentist is a student not only when he is acquiring the rudiments of his art; he is always a student, nor can he ever hope to get beyond the contracted circle that bounds his very imperfect ideas, if when he nails up his sign, he packs away his books, and shuns the place where wisdom is to be gained.

*When a Dentist ceases to study, he should cease to practice.*

The mental progress of a studious Dentist may be illustrated by a succession of circles each of greater diamater than that which precedes it. With limited knowledge he enters the first, fills it and passes into a larger; effort and expanse of thought soon find this limit too small, and he presses on into another, with wider bounderies. Thus onward he goes, taking broader views of things, enlarging his sphere of action, extending his usefulness, growing into a more and more perfect man, never content with present attainment but ever reaching out for higher.

Such a course of studious devotion to the science which he has chosen as his life's pursuit, will secure a thoroughness of acquaintance with it, that will fit him for the performance of his duty towards it; and without this he is not fitted for that duty, nor ever can be.

When the Dentist thus thoroughly knows his profession and what it is capable of becoming, he is prepared to labor intelligently for its elevation, which he should do; first, *in point of efficiency.*

His aim should be to bring up his art to that point of excellence, where it can do the most good; and where it can do good of so marked and unquestionable a character, as to command attention, and make itself felt among the professions of the world.

To do this, he must employ not only his hands; his mental faculties must also bend to the task. And there will be periods in his life, when *hours* must be passed in the severest mental toil, in order to secure a single *step's* advance. But for his encouragement, let him remember this:—As in a day's journey, (which is made up of single steps) each *one* is of essential importance, so, in traveling along the difficult path of scientific progress, each smallest step, each *iota* of advance, becomes of untold value;—for, it, by just so much, leads on toward the desired end, and may be the key to the door of difficulty, which, opened, shall introduce us to the treasures beyond.

A Dentist has this advantage over many who pursue scientific research. He can work out with his fingers, the theories he has wrought out in his brain, and prove their practicability. His hands driven by his mind, his mind energized and kept in even balance by scrutiny of his handiwork.

Mental labor and manual labor;—these, the means.

The increased efficiency of the profession;—this, the end.

The faithful Dentist should seek to elevate his profession not only in point of efficiency, but also in point of respectability. It is high time that the public were made to regard Dentistry as a "*learned* profession;" and not as a simple *trade*, which can be "taken up" by any man of ingenuity, without preparation or study.

The Dentist who loves his profession is jealous of its reputation. And the more thorough his knowledge of it, the more sensitive will he be to the estimation in which it is held.

I have had the remark made to me by a gentleman, who heard of some charge of mine which he considered high: "Well, I'll leave the dry goods trade and go into Dentistry," accompanied by an expletive, more forcible than pious.

"You would have to work eight or ten years sir, before you could *do* anything if you did," was the indignant reply.

There has been too much of this low estimate of the Dental art abroad in the land, and every practitioner should do what he can to raise the standard of his profession, and compel people to accord it that respect which is its due.

It is a great satisfaction to intelligent men to feel, that they are able to do some things which everybody can not do; and a satisfaction, only second to this, is in compelling people to acknowledge it. Now people are both gullible and obstinate; and my observation has led me to the conclusion that just in proportion as an art or a profession is *useful*, just in that proportion are people *obstinate* in acknowledging its merits.

Jugglery finds its disciples readily.

A mountebank has little difficulty in securing supporters. The brazen-faced fortune teller has but to exhibit her sign, and scores of hands are held out for her scrutiny, each containing its silvery passport to her favor. But he who offers his services to do a real good, must toil for years to build up a reputation that will afford him support. But, the *true* man finds in this fact no encouragement to become a mountebank in his profession. Patiently, assiduously, he devotes himself to his pursuit, in the full determination to stand before the world a full fledged man worthy of its confidence and support, or not stand before it at all.

Nor will he fail, if such be his purpose. Knowledge and efficiency will command respect, and the pursuit in connection with which, such knowledge and efficiency are displayed, must necessarily rise in public estimation.

There are three classes of motives to actuate the Dentist in his efforts to elevate his profession.

The first or lowest of these, is drawn from the consideration of self-interest.

This is, undoubtedly, the most powerful motive to action among men, if considered in the aggregate as to its results.

Of itself it is sufficient to induce the Dentist to labor for the elevation of his profession. For, when his profession rises, he rises with it, and takes a larger share of public favor in proportion as a more favorable regard is extended to it.

Every man is pleased with the good opinion of his fellow men, and when that good opinion is accompanied by pecuniary tokens of its strength, it is doubly gratifying.

Therefore, without enlarging more on this point, it is evident that even this motive of self interest, unworthy though it be, is found on the side of the Dentist who aims at elevating his profession.

The second motive, impelling in the same direction, proceeds from the principle of gratitude.

The Dentist's profession gives him a standing, gives him a support, gives him the means and opportunity of improvement and growth. In return he should give to it his earnest efforts, not for the sole purpose of "making it pay better," but of making it stand higher. It takes care of him, let him take care of it. It gives him sustenance, let him give it character. The commonest principle of gratitude to benefactors, prompts to the making some return for favors received. Let the Dentist then, seek to make the only return to the profession which supports him, that he can make, viz.: to elevate its character by every means in his power.

The third motive is drawn from the consideration of *honor.* A noble profession has been committed to his hands. Let honor prompt him to guard well its interests. He is accepted among the fraternity who have chosen the same branch of scientific industry as one of them. Honor would urge him to shun any course of action calculated to bring discredit upon that fraternity. More than this. Honor would incite him to such action as will bring positive credit upon them, to employ every means calculated to advance his art, that he be not found recreant to his duty. A growing science claims help from *all* its adherents, that its growth may not be checked nor its progress stayed, short of full and perfect development. And none *can*, in honor, withhold his mite of effort. A faithful, honorable devotion to the profession of his choice, will impel every Dentist to join with earnest heart and active hand, in whatever tends to increase its efficiency and its reputation.

Of course, it is impossible to do more than glance at the several topics suggested by this almost exhaustless subject, in the space of a single paper, and having cursorily examined the first question, I ask your attention while I more briefly consider the second.

2d. How does the Dentist's duty to his profession affect other duties in his life?

It is argued by some, that the Dentist's duty to his profession is paramount to all other duties, or at least, such would be the natural inference from remarks that have been made in this Association.

Now is this true? Is the practitioner of Dentistry to feel, that in order to secure the plaudit, "well done, good and faithful one," he must neglect all other duties, and devote himself exclusively to those growing out of his profession?

And if he ventures to bestow some attention upon other duties, must he expect to rest under the charge of remissness and culpable neglect ?

I answer most emphatically, *No !*

Life is made up of duties, various in their character, and varied in their object. Nor can a man neglect *any* duty, without breaking a link in that chain of consistent action which should characterize his life. The importance of these duties, depends upon the importance and character of the objects to be attained by their performance. The great Maker of us all, has given us our spheres of action, and has assigned to each of us our respective duties in those spheres. If we select one, and neglect the rest, though we discharge that one most faithfully, we fail grandly ; especially is this so, if we lose sight of the greater and choose the less.

It has been said here that this Association is the best prayer meeting a Dentist can attend. I repudiate such a doctrine. I do not so think, and my conscience will not let me allow its annunciation to go unrebuked.

This association is good and I love it. We get great good by coming here, and what I have said in the first part of this paper shows clearly what my mind is on this point. The Dentist has a great duty resting upon him in regard to his profession, the duty of study, of investigation, in a word, the duty of perfecting himself to the highest degree possible in his art. But this is not his only duty in life. He has others, and some far above this, as far above it as the twinkling lights in yonder heavens, are above the jets of illumination which meet our eyes at this moment ; as far surpassing it in magnitude as each one of these worlds which appears to us but as an eye of light, surpasses in reality of magnitude, the insignificant globe that shades our midnight lamp.

The faithful performance of secular duty is accepted by the religious man as obligatory upon him. He feels that it is right he should do it ; but it never will be accepted by the Supreme Master *in the place* of religious duty. They must be kept distinct. And as a man's *soul* is worth more than a man's *teeth*, so are those observances which affect the soul's welfare, of first moment.

The true idea is this; the harmonious arrangement of all duties, secular and religious, social, domestic and public; each in its place, none allowed to override the other, but all receiving the meed of attention and of faithful observance to which they are entitled. Such should be our standard.

So shall we be not men of one idea, men of one duty, rushing at break neck pace along a single track, but well-balanced, consistent, well-developed *men*, who look at things with a clear vision, and are able to discriminate between what is and what is not duty, in every situation of life; one not judging for all, but each for himself. Let us then be earnest in duty, persevering in effort, aiming high, bringing all our moral principle, all our religious principle, into the liveliest action, and making them bear upon whatever we undertake that our action may be pure, and our success, if success be ours, be characterized by unmingled virtue.

